# Sarcomatous Tumors of the Jaws

**Published:** 1876-10

**Authors:** J. Ewing Mears


### ARTICLE IV.

### *Sarcomatous Tumors of the Jaws.*

One involving the masseter muscle; a second of the parotid gland, involving the masseter muscle, both removed by operation, were reported by J. Ewing Mears, M. D.

The first case was one of peripheral osteo-sarcoma, taking its origin in the periosteum of the lower jaw, in a boy twelve years of age, a resident of Delaware, who was brought to the clinic at the Pennsylvania College of Dental Surgery, in the month of October, 1873. The following history with regard to the cause of the tumor was obtained at that time. In the month of July preceding, whilst in bathing, he received a severe blow upon the left side of the face over the region of the lower jaw, caused by coming in contact with the head of one of his comrades as he sprang into the water from the pier. At the time of the receipt of the injury he experienced considerable pain, and soon after swelling supervened. The soreness in part gradually diminished and finally disappeared, leaving, however, a slight enlargement. This was so slight as to produce no deformity, and in a few weeks the accident and its results were dismissed from mind.

During the course of the summer attention was attracted to a swelling of the face, which was attributed to a suspected decayed tooth which had been attacked by cold. The usual remedies employed in such cases were applied without causing the disappearance of the swelling, and the boy was placed under the care of a physician in the town in which he resided. At first the physician was disposed to regard the swelling as due to the cause ascribed by the members of the family, and directed treatment accordingly, Failing to obtain the results expected from the treatment, he was led to make a more careful and extended examination, and arrived at the conclusion that the case was of a more serious nature; he then advised that the patient should be brought to the city and placed under the care of a surgeon.

On examination, the left side of the face was found very much enlarged, the swelling occupying more particularly the region of the lower jaw, the greater portion of the body the angle and ramus. To the touch it was hard, dense, and inelastic, and firmly attached to the bone. The overlying tissues were freely movable and yielding. The finger introduced between the cheek and the teeth into the buccal space could distinctly trace the attachment of the growth to the lower jaw. The external surface of the upper jaw was normal and the canine fossa intact, showing the antrum to be free from disease. There was almost entire occlusion of the jaws, the patient being able by great effort to separate them to the extent of but one-quarter of an inch. In order to examine the internal surface of the lower jaw it was necessary to give the patient ether and forcibly separate the jaws by a lever, when the internal surface was found normal and not in any way affected by tumor. The introduction of the exploring needle into the mass from the inside was followed by any escape of blood.

At no time during the development of the growth had pain been a prominent symptom. The increase in size had been very rapid, and the occlusion of the jaws compelled the patient to confine himself to the ingestion of liquid food. His general health was not notably impaired.

Owing to the closure of the jaws, it was found impracticable to effect removal by an internal incision, beginning just below the lobule of the ear, carrying it downward to the angle of the jaw, along the posterior border to a point within one inch of the symphysis. The facial artery was secured at the anterior inferior angle of the masseter muscle where it mounts upon the jaw, and divided. Dissecting the skin and superficial facia upward to some distance, a freely movable flap was formed, which, on being raised, exposed the upper portion of the tumor. In order to reach its attachments below, it was found necessary to detach the superficial structures to some extent into the region of the neck posteriorly. The masseter muscle at its insertion was incorporated in the tumor; above, to one-third of its extent it was free, and, on being divided, retracted to its points of origin. Having fully exposed the tumor, an effort was made to separate it from the jaw by the knife; but this was found impossible, and recourse was had to the chisel, by which it was detached, removing with it the external plate of the bone, softened and disintegrated to the extent of attachment of the tumor; posteriorly, the growth passed over the border of the ramus, and penetrated the deeper structures of the neck, overlying the external carotid artery this portion was removed by careful dissection, and the rough spiculated surface of the bone was thoroughly scraped by the chisel.

The second case was one of sarcoma of the parotid gland of the right side, occurring in a patient who was admitted into the surgical wards of St. Mary's Hospital, in the summer of the year 1874, for a swelling which occupied the right side of his face.

He was fifty-eight years age, and a furrier by trade. He had enjoyed good health until two years ago, when had a partial attack of paralysis, involving the right side. This condition interfered somewhat with his work, but was not sufficient to compel him to abandon it. Three months prior to his admission he noticed a slight swelling upon the right side of the face, aud at the same time experienced difficulty in opening his mouth to the usual extent. The swelling increased quite rapidly, and with its increase in size the occlusion of the jaws became more marked. A physician was consulted, who regarding it as an inflammatory swelling, ordered sorbefacient applications. No benefit appeared to be derived from the treatment pursued, and the patient came to the hospital.

At the time of his admission the tumor had grown to quite a large size, and the occlusion of the jaws was nearly complete. An examination revealed the fact that the parotid gland and the masseter muscle were involved, and the jaw itself was supposed to be implicated. the removal of the growth having been determined upon, it was exposed by an incision extending from the zygoma downward, in front of the ear, and along the border of the ramus and body of the jaw to within an inch and a half of the symphysis.

The flaps formed by this incision weie freely dissected upward, upon the cheek, and downward, upon the neck, and the tumor, consisting of the parotid gland and the entire masseter muscle, was removed. The facial and external carotid arteries were in turn ligated—the facial before, and the external carotid artery after division. The jaw was found to be free from disease. The wound was closed by iron-wire sutures, and a compress and bandage were applied. Considerable suppuration ensued before the wound finally healed, which occurred four weeks after the operation.—*Philadelphia College of Physicians and Surgeons.—Med. and Surgical Reporter.*

---

### ARTICLE V.

### *Odontological Society of Great Britain.*

